# Primary prevention of cardiotoxicity in cancer patients treated with fluoropyrimidines: a randomized controlled trial

**DOI:** 10.1186/s40959-025-00344-3

**Published:** 2025-05-17

**Authors:** Johanne D. Lyhne, Vibeke B. Hansen, Lone D. Vestergaard, Susanne E. Hosbond, Martin Busk, Mayooran Gnanaganesh, Else Maae, Birgitte M. Havelund, Torben F. Hansen, Signe Timm, Lars H. Jensen, Mads D. Lyhne

**Affiliations:** 1 Department of Oncology, Lillebaelt Hospital –Vejle, University Hospital of Southern Denmark, Beriderbakken 4, Vejle, 7100 Denmark; 2 Department of Cardiology, Vejle Hospital, University Hospital of Southern Denmark, Vejle, Denmark; 3 Department of Clinical Medicine, Aarhus University, Aarhus, Denmark; 4 Department of Anesthesiology and Intensive Care, Aarhus University Hospital, Aarhus, Denmark

**Keywords:** Cardio-oncology, Cardioprotection, Coronary artery calcium score, Adverse effects, 5-FU, Capecitabine

## Abstract

**Background:**

Fluoropyrimidines (FP) are the third most used chemotherapeutic drugs administered in solid tumors but have cardiotoxic side effects. We aimed to determine whether pre-chemotherapeutic cardiological assessment and management of cardiovascular risk factors could prevent FP-induced cardiotoxicity and if the coronary artery calcium (CAC) score was predictive of chest pain.

**Methods:**

This was a randomized, controlled, single center trial of patients with various cancer types who were treated with FP and had no known ischemic heart disease. All patients had CAC score obtained by cardiac CT scan. Patients were randomized to pre-chemotherapeutic cardiological management or standard care. Cardiological management included risk reduction based on electro- and echocardiographic evaluation and blood samples. Primary composite endpoint included hospital admission for chest pain, acute coronary syndrome, coronary angiography intervention, or all-cause mortality. Secondary outcome was chest pain. Follow-up was 6 months. Data were analyzed using Kaplan–Meier survival function with log-rank test and ROC-analyses.

**Results:**

Of the 192 patients included, the primary endpoint occurred in 9/95 (9.5%) patients in the intervention group and 15/97 (15.5%) patients in the control group (log-rank *p* = 0.19) with an incidence rate ratio (IRR) of 0.57 (95% CI [0.22 – 1.39]). Chest pain occurred in 6/95 (6.3%) patients in the intervention group and 13/97 (13.4%) in the control group, yielding an IRR of 0.44 (95% CI [0.14 – 1.23]). CAC score did not predict chest pain occurrence.

**Conclusions:**

Cardiological management of cardiovascular risk factors prior to treatment with fluoropyrimidines resulted in half as many cardiotoxic events but the study did not reach statistical significance**.** Further studies are needed to investigate the optimal strategies to prevent fluoropyrimidine-induced cardiotoxicity in cancer patients.

**Trial registration:**

ClinicalTrials.gov Identifyer NCT03486340.

**Supplementary Information:**

The online version contains supplementary material available at 10.1186/s40959-025-00344-3.

## Background

Fluoropyrimidines (FPs) are highly effective and rank as the third most frequently administered chemotherapeutic agents in patients with solid tumors [1, 2]. FPs have known side effects, including cardiotoxicity [1, 3] with the majority of events occurring within the first 72 h after chemotherapeutic administration [4]. Fluoropyrimidine-induced cardiotoxicity occur in approximately 5% of patients [5].

FP-induced cardiotoxicity often manifests as chest pain which suggests the underlying mechanisms target coronary arteries [6, 7]. Cardiac CT is a frequently used modality for investigation of coronary artery disease with the coronary artery calcium (CAC) score associated with increased risk of cardiovascular events [8]. Given this, the CAC score has been proposed as a potential tool for risk stratification [9], also in cancer patients [10]. Several cardiovascular risk factors have been associated with higher rates of FP-induced cardiotoxicities, although inconsistently [11–14].

Although FPs are recognized as cardiotoxic agents, current cardio-oncology guidelines and statements provide limited information on FP-induced cardiotoxicity [6, 15–17], and studies have focused on management of manifest cardiotoxic events rather than prevention [1]. Studies designed to prevent cardiotoxicity have mainly been conducted in patients receiving anthracyclines and/or human epidermal growth factor receptor 2–targeted therapies [18, 19]. There is an evident need to investigate the potential of preventing FP-induced cardiotoxicity.

Our study aimed to explore the feasibility of preventing FP-induced cardiotoxicity through early cardiological screening and intervention. We hypothesized that pre-chemotherapeutic management of cardiovascular risk factors could reduce FP-induced cardiotoxicity. Furthermore, we hypothesized that a high CAC score would be associated with higher incidence of FP-induced chest pain.

## Methods

### Ethics

The study complied with the Declaration of Helsinki and was ethically approved by the The Regional Committee on Health Research Ethics for Southern Denmark (S-20170132). All patients gave oral and written consent to participate in the study. The study was prospectively registered at clinicaltrials.gov (NCT03486340).

### Study design

This prospective 1:1 randomized controlled trial was conducted and reported in accordance with the CONSORT guidelines. The study was designed to compare standard oncological assessment without formal cardiological risk stratification to a proactive cardiological evaluation and risk factor management strategy prior to FP treatment, regardless of prior exposures or known cardiovascular risk factors.

Participants were randomized to intervention or control group. Randomization was performed by a computer-generated random table (REDCap) with no restrictions. Before initiation of FP treatment, all included participants underwent non-contrast cardiac CT for CAC measurement, blood pressure measurement, electrocardiogram (ECG), and routine laboratory tests for creatinine, haemoglobinA1c (HbA1c), and lipids. Comorbidities (diabetes, hypertension, hypercholesterolemia) and smoking status were recorded. For study schedule, see Table 1 in supplementary.

The intervention group was referred to further cardiologic assessment within few days, which included transthoracic echocardiography (TTE) by specialized cardiologists. Aggressive interventions were initiated according to existing guidelines [20, 21], see Table 1. Treatment decisions (e.g., choice of drug) were left to the discretion of the treating cardiologist and were guided by the patient’s overall clinical profile. Patients were encouraged to smoking cessation, healthy lifestyle initiatives and referred to their general practitioner if relevant. The control group was not referred to further cardiologic assessment or interventions before oncologic treatment, and their CAC result remained blinded until the conclusion of study period. The follow-up period was six months to encompass the standard FP treatment period identifying acute cardiotoxic events occurring during or shortly after chemotherapy administration.

**Table 1:** Cardiological management of risk factors

Indication	Intervention
Calcium score above the age- and gender-adjusted median and/or CAC score > 400	Prophylactic daily aspirin 75 mg and statins (atorvastatin 40 mg daily), if tolerated. If already on statins, increase the dose if possible
LDL cholesterol above 3 mmol/l	Statins (atorvastatin 40 mg daily), if tolerated. If already on statins, increase the dose if possible
LDL cholesterol above 3 mmol/l already receiving 80 mg atorvastatin	Supplement with ezetimib (10 mg daily)
LDL cholesterol above 3 mmol/l already receiving 80 mg atorvastatin and ezetimib	Switch to rosuvastatin (40 mg daily)
Hypertension (two measurements above 140/90 mmHg)	Perform 24-h ambulatory blood pressure monitoring. If hypertension is confirmed, initiate treatment according to guidelines
HbA1c > 48 mmol/mol	Refer to further evaluation and treatment by the general practitioner
Lifestyle risk factors	Educate on interventions according to guidelines (e.g., smoking cessation, regular exercise, nutritious diet)
Abnormal echocardiography	Handle according to findings and guidelines
Chest pain + risk factors ± ECG changes	Refer to coronary angiography

Interventions according to existing guidelines. *Abbreviations*: *CAC* coronary artery calcium, *LDL* low-density lipoprotein, *HbA1c* hemoglobin A1c, *ECG* electrocardiogram

### Study population

Eligible patients were enrolled from the outpatient clinic at the Department of Oncology, Vejle Hospital, Denmark between April 2018 and December 2023. Patient enrolment was temporarily paused and consequently delayed during the COVID-19 pandemic and again during the Danish national nurse’s strike in 2021.

Eligible patients were aged 18 years or above with a histologically confirmed cancer diagnosis and clinical indication for (neo)adjuvant or palliative treatment with FP (5-FU or capecitabine). Patients who had previously been treated with FP were excluded. To ensure that only asymptomatic patients without clinically apparent cardiac disease were included, patients were also excluded if they had known ischemic heart disease, ischemia-suspicious symptoms prior to FP treatment or ischemia-suspicious ECG-changes.

### Hypotheses and endpoints

We listed a priori three hypotheses with concomitant endpoints:

1. In cardiac asymptomatic patients, management of cardiovascular risk factors before initiating FP treatment will reduce the incidence of Major Adverse Cardiovascular Events (MACE) defined by: Hospital admission for chest pain, non-ST-elevation myocardial infarction (NSTEMI), ST-elevation myocardial infarction (STEMI), coronary angiography intervention, acute coronary syndrome (ACS) including unstable angina pectoris (UAP), and all-cause mortality during six months post-randomization compared to the control group (*primary endpoint*).
2. In cardiac asymptomatic patients, management of cardiovascular risk factors before initiating FP treatment will reduce the incidence of chest pain during six months post-randomization compared to the control group (*secondary endpoint*).
3. A CAC score above the age- and gender-adjusted median (50), CAC > 400 and/or as a continuous variable can identify patients at increased risk of developing chest pain during FP-treatment (*tertiary endpoint*).

### Cardiac CT and CAC score

The CAC-score was assessed on a non-contrast cardiac CT scan on a dual-source CT scanner (SOMATOM Definition Flash, Siemens Healthcare, Forchheim, Germany) with prospective ECG triggering at 65–75% of cardiac R-R interval. The scanning parameters were as follows: slice thickness 3 mm, collimation 128 × 0.6 mm, gantry rotation time 0.28 ms, 120 kV tube voltage, and 90 mAs/rotation. The cardiac-CT scan was performed during inspiratory breath hold and without pre-examination treatment with a beta-blocker. Post-processing was conducted on a dedicated workstation using Syngovia (Siemens).

According to the Agatston method [22], the CAC score quantifies the extent of overall coronary atherosclerosis as Hounsfield units multiplied by the area of the calcification in square millimetres and was evaluated by specialized cardiologists. The scale score ranges from 0 to > 400 with the following categorization: 0: normal; 1 – 10: discrete coronary atherosclerosis; 11–100: mild coronary atherosclerosis; 101–400: moderate coronary atherosclerosis; and > 400: severe coronary atherosclerosis. CAC scores were measured both as an absolute value as well as an age- and gender-adjusted percentiles compared to background population data [23]. A CAC score > 400 or > 50th percentile was defined as indicative of increased coronary atherosclerosis requiring treatment (Table 1) [24].

### Events

As part of standard oncological practice, all participants were informed about the potential cardiac side effects associated with FP treatment by their oncologist and instructed to promptly contact the Department of Oncology if they experienced any cardiac symptoms (classified as “events”). If clinically indicated, patients were referred to the Department of Cardiology for further examination and treatment.

In case of an event, cardiac symptoms (chest pain, dyspnea, tiredness, or palpitation) were registered. Chest pain was categorized as typical stable angina (characterized by pressing chest pain exacerbated with exercise and relieved with rest or nitroglycerine), unstable angina (characterized by pressing chest pain occurring with minimal or no exertion), or atypical chest pain.

Additionally, the presence of ECG changes and the results of laboratory tests (hemoglobin, TnT and CRP) were recorded, as was the need for any interventions, such as coronary arteriography (CAG) or percutaneous coronary intervention (PCI). The urgency of the situation was determined by the treating cardiologist and categorized as either hospital admission or outpatient treatment (subacute or elective).

Follow-up assessments were conducted for both intervention and control group six months after initiating FP treatment at the Department of Oncology. Each follow-up session included a physical examination, ECG, blood pressure measurements, routine laboratory tests and questions addressing any cardiac symptoms not reported during the six-months intervention period. The primary endpoint was evaluated through patient records review after study completion.

### Statistical analyses

An a priori power analysis was conducted with a β-value of 0.2 (power = 0.8) and an α-value of 0.05, indicating that a total of *n* = 398 participants would be required to detect a 50% reduction in the presence of MACE at follow-up (from 20 to 10%). The study was initially planned with an interim analysis after a two-year inclusion period with an expected sample size of 200 patients. However, due to the described significant enrolment delay caused by the COVID-19 pandemic and a national nurse strike, the study was terminated at the interim time point. Given this, it was anticipated that the study would be underpowered to detect a statistically significant difference. Descriptive statistics was used to summarize patient demographic and clinical characteristics by reporting mean with standard deviations (SD) and medians with interquartile range as appropriate for continuous measures, and absolute numbers and proportions (%) on nominal and binary measures. Standardized differences were calculated to investigate baseline exchangeability between the intervention group and control group, and an absolute standardized difference of > 0.2 was considered imbalance [25].

Occurrence of the primary endpoint (MACE) was analyzed using Kaplan–Meier survival function defined as the time from randomization to the time of MACE (whichever event occurred first). The survival function in the two groups was compared using log-rank test, and likewise prevalence of the MACE was compared using Chi^2^ test. Sensitivity analyses included investigation of a redefined MACE substituting overall mortality with cardiac related death, and per-protocol analyses taking cross-over of non-compliant patients into account (*n* = 2). Incidence rates were reported as incidence per 1,000 person-years and as incidence rate ratios (IRR) with 95% CI, comparing the occurrence of primary and secondary outcomes in the two groups.

The discriminatory properties of CAC-score to identify patients with hospitalization for chest pain during the first six months was investigated using Receiver-Operating Characteristic (ROC) analyses. CAC-score was investigated in the following four categorizations and compared using Area Under the Curve (AUC): 1) CAC above or below 400, 2) age- and gender adjusted CAC-score above or below the median (50), 3) categorical CAC (0: normal; 1 – 10: discrete coronary atherosclerosis; 11–100: mild coronary atherosclerosis; 101–400: moderate coronary atherosclerosis; and > 400: severe coronary atherosclerosis), and 4) continuous CAC. Analyses were performed on all patients and sensitivity analyses were performed excluding patients undergoing CAC-reduced treatment as part of the intervention.

Post-hoc analyses included a paired t-test comparing LDL-cholesterol at baseline and six months follow-up to investigate indications of compliance among patients undergoing cholesterol reducing treatment, and explorative stratification analyses on the incidence rate of MACE based on cardiovascular risk factors.

All statistical testing were performed two-sided and *p*-values < 0.05 were considered statistically significant. Statistical analyses were performed using STATA version 18.

## Results

**Fig. 1:**
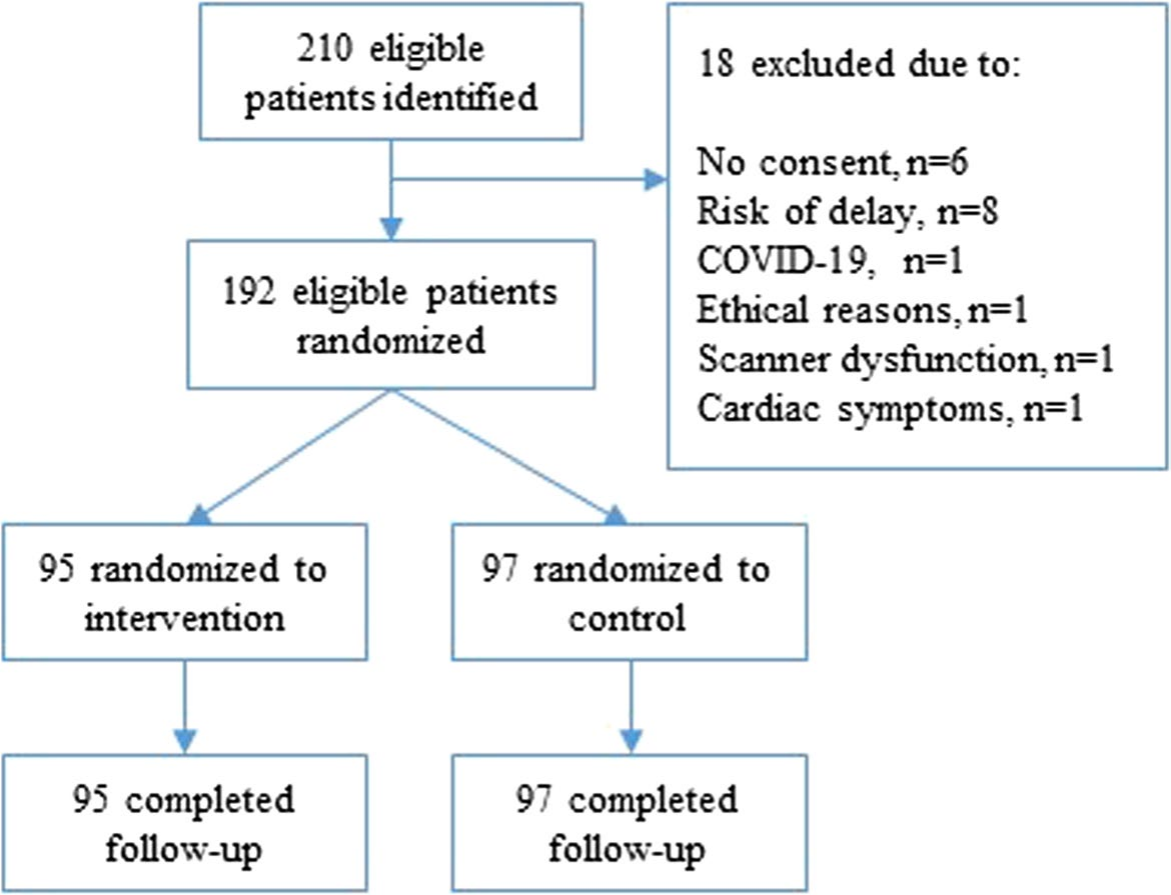
CONSORT flow diagram

Two hundred and ten patients were enrolled in the study. Eighteen (8.5%) were excluded due to withdrawal of consent (*n* = 6), risk of delaying the initiation of chemotherapy treatment (*n* = 8), COVID-19 (*n* = 1), ethical regulations (*n* = 1), CT scanner dysfunction (*n* = 1) and existing cardiac symptoms (*n* = 1). Accordingly, 192 patients were included in the study of which 95 were randomized to intervention group and 97 to the control group, see Fig. 1. Baseline demographic, clinical and laboratory characteristics of the two groups were comparable, see Tables 2 and 3. No participants were lost to follow-up.

**Table 2:** Patient characteristics

Characteristics	Total (*n* = 192)	Intervention group (*n* = 95)	Control group (*n* = 97)	Standardized difference
Age, years	65 (58–72)	65 (59–73)	66 (58–71)	0.08
Male sex	99 (51.6)	51 (53.7)	48 (49.5)	0.08
Cardiovascular risk factors				
BMI, kg/m^2^	27.2 (4.8)	27.9 (4.6)	26.4 (4.9)	-0.31
Predisposed to CVD	44 (22.9)	18 (18.9)	26 (26.8)	0.23
Known EF < 45%	3 (1.6)	1 (1.1)	2 (2.1)	N/A
Aspirin treatment	19 (9.9)	11 (11.6)	8 (8.2)	0.11
Diabetes	31 (16.1)	13 (13.7)	18 (18.6)	0.13
Hypertension	89 (46.4)	47 (49.5)	42 (43.3)	0.12
Hypercholesterolemia	56 (29.2)	25 (26.3)	31 (32.0)	0.12
Active smoker	31 (16.1)	14 (14.7)	17 (17.5)	0.08
Cancer characteristics				
Site				
Colon	93 (48.4)	43 (45.3)	50 (51.5)	0.28
Rectum	41 (21.4)	24 (25.3)	17 (17.5)	
Pancreas	19 (9.9)	7 (7.4)	12 (12.4)	
Breast	36 (18.8)	20 (21.1)	16 (16.5)	
Appendix	3 (1.6)	1 (1.1)	2 (2.1)	
Stage				
I	5 (2.6)	1 (1.1)	4 (4.1)	0.39
II	16 (8.3)	9 (9.5)	7 (7.2)	
III	74 (38.5)	33 (34.7)	41 (42.3)	
IV	91 (47.4)	47 (49.5)	44 (45.4)	
Missing	6 (3.1)	5 (5.3)	1 (1.0)	
Treatment indication				
Adjuvant	97 (50.5)	48 (50.5)	49 (50.5)	0.02
Neo-adjuvant	41 (21.4)	20 (21.1)	21 (21.6)	
Palliation	54 (28.1)	27 (28.4)	27 (27.8)	
Treatment regimen				
Capecitabine monotherapy	79 (41.1)	45 (47.4)	34 (35.1)	0.25
Capecitabine in combination^a^	80 (41.7)	35 (36.8)	45 (46.4)	
5-FU in combination^b^	33 (17.2)	15 (15.8)	18 (18.6)	
Concomitant radiation	6 (3.1)	4 (4.2)	2 (2.1)	0.12
Previous anthracycline treatment	29 (15.1)	15 (15.8)	14 (14.4)	0.15
Previous chest radiation	31 (16.1)	16 (17.0)	15 (15.5)	0.15

Caption: Demographics, cardiovascular risk factors and cancer characteristics for all patients and stratified by randomization group. Data are presented as mean (SD), median [IQR] or n(%) as appropriate. ^a^In combination with oxaliplatin, irinotecan, transtuzumab, or other. ^b^In combination with oxaliplatin, irinotecan, cetuximab/manitumumab, or orther

*Abbreviations*: *BMI* body mass index, *CVD* cardiovascular disease, *EF* ejection fraction

**Table 3:** Paraclinical characteristics

	Total (*n* = 192)	Intervention group (*n* = 95)	Control group (*n* = 97)	Standardized difference
CAC score, median (IQR)	26 (0 – 214.5)	17 (0 – 211)	43 (0 – 234)	N/A
Adjusted CAC score, median (IQR)	42 (0 – 78)	39 (0 – 68)	55 (0 – 80)	N/A
CAC score > 400	29	14	15	0.02
Systolic blood pressure, mmHg	137 (18)	137 (17)	138 (19)	0.07
Heart rate, bpm	78 (15)	77 (12)	78 (17)	0.07
Creatinine, mg/dL	76 (21)	76 (21)	75 (20)	-0.04
HbA1c, mmol/mol	40 (9)	39 (8)	41 (9)	0.22
Lipid profile, mmol/L				
Cholesterol	4.8 (1.1)	4.8 (1.1)	4.8 (1.1)	-0.02
HDL	1.4 (0.4)	1.4 (0.4)	1.4 (0.5)	0.02
LDL	3.1 (1.0)	3.1 (1.1)	3.0 (1.0)	-0.06
Triglyceride	1.9 (1.1)	1.9 (1.0)	1.9 (1.1)	-0.04

Baseline CAC score, blood pressure and heart rate and laboratory data for all patients and stratified by randomization group. Data are presented as mean (SD) or median [IQR] as appropriate

*Abbreviations: CAC* Coronary artery calcium, *HbA1c* hemoglobin A1c, *HDL* High-density lipoprotein, *LDL* Low-density lipoprotein

In the intervention group, 88 patients (92.6%) underwent at least one cardiological interventions before start of FP treatment. The most frequent interventions were lifestyle risk factors information (*n* = 57) and statin prescription (*n* = 43). Twenty-eight patients received both aspirin and statin due to an elevated CAC score, and 14 patients with elevated blood pressure were prescribed antihypertensive medication. Six patients were referred to their general practitioner for blood glucose control management. Guideline-based treatments were initiated for abnormal TTE findings (*n* = 7) and ischemia tests (*n* = 3).

At follow-up, we observed a significant decrease in LDL for patients in the intervention group with a statin prescription from 4.0 to 2.7 mmol/L (difference -1.3, 95% CI [-1.7; -0.9], *p* < 0.001) indicating compliance.

### Prevention of primary, composite endpoint

**Fig. 2:**
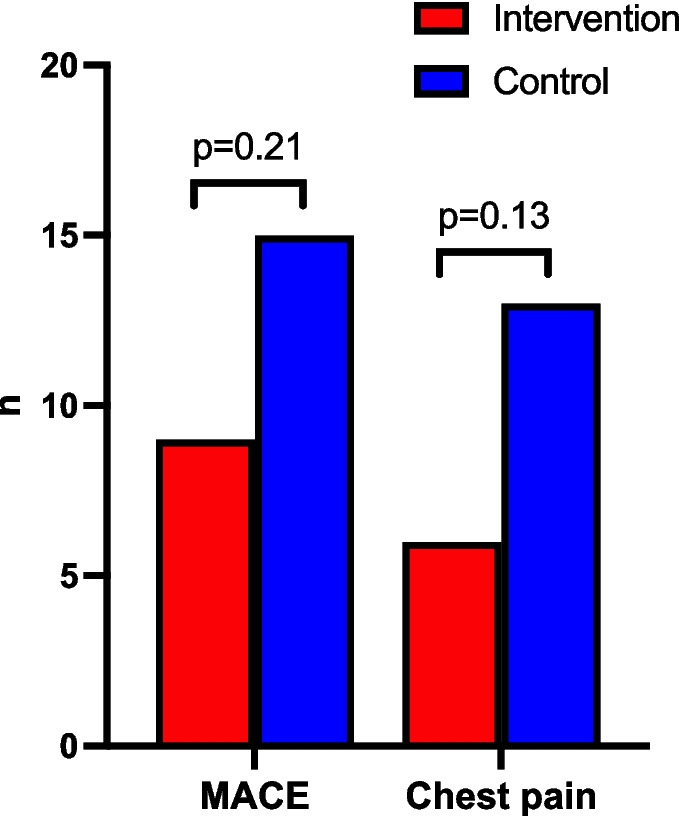
Prevalence of events. Caption: Prevalence of major adverse cardiovascular events (MACE, primary outcome) and chest pain (secondary outcome) in the intervention group and control group during 6 months’ follow-up following fluoropyrimidines treatment

The MACE occurred in 24 patients (12.5%) with 9/95 (9.5%) patients in the intervention group and 15/97 (15.5%) patients in the control group, see Fig. 2. Hospital admission was needed in 10 cases, including two classified as ACS (both in the intervention group), for which CAG was performed. One of the patients had STEMI in the ECG but completely normal CAG. The other patient had low grade atherosclerosis on the CAG but no stenoses and no need for revascularization. FP-induced vasospasm was suspected in both ACS cases and these patients were subsequently withdrawn from further FP treatment. During the study period, 14 patients (7.3%) died, with the majority (*n* = 12, 85.7%) due to cancer. Of notice, the remaining two died from cardiac causes and were both in the control group. For further details see Table 2 in supplementary. Additionally, following a MACE, two patients in the control group were withdrawn from FP treatment upon the decision of the treating oncologist. No important harms or unintended effects were observed.

**Fig. 3:**
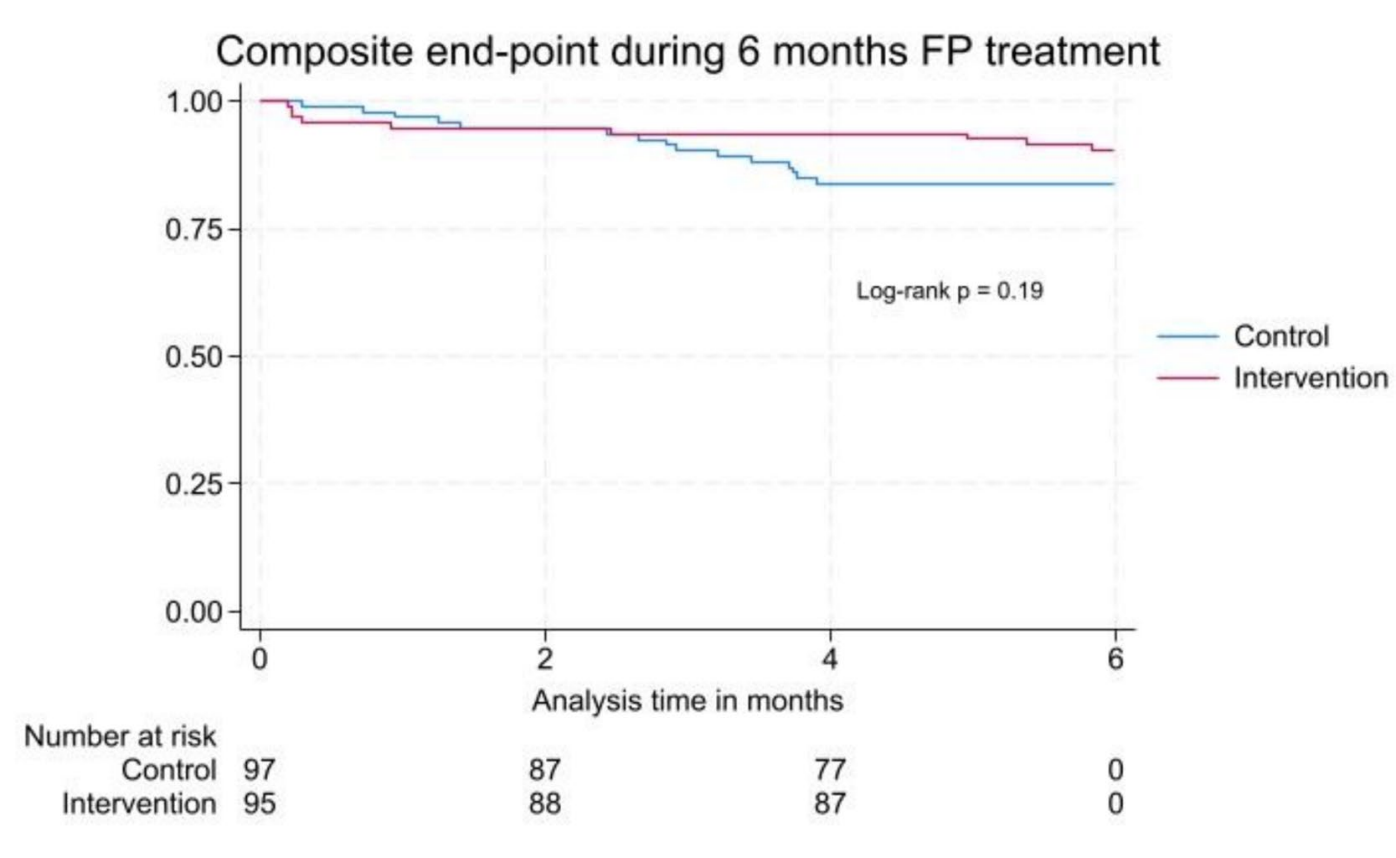
Kaplan–Meier plot**.** Kaplan–Meier survival function defined as the time from randomization to the time of the major adverse cardiac endpoint (primary outcome) in the intervention group and control group during 6 months’ follow-up following fluoropyrimidines treatment

**Fig. 4:**
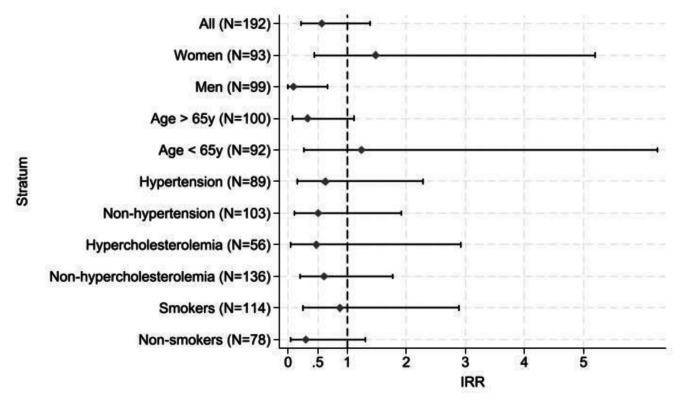
Exploratory stratification analyses on the MACE based on cardiovascular risk factors**.** Caption: Stratification analyses of the incidence rate of major adverse cardiac events (MACE) based on established cardiovascular risk factors: Age, sex, hypertension, hypercholesterolemia, and smoking status. Diabetes and predisposition to cardiovascular events were not included in stratification analyses due low incidence of MACE

The Kaplan–Meier survival plot showed similar incidence of MACE in the two groups (Log-rank test *p* = 0.19), see Fig. 3. The incidence rate of MACE in the intervention group was 205 events per 1,000 person-years and 359 events per 1,000 person-years in the control group resulting in an incidence rate ratio (IRR) of 0.57 (95% CI [0.22 – 1.39]). Sensitivity analysis on cardiac related death did not change the result, neither did per protocol analysis with cross over of two patients not receiving statins though indicated (data not shown). Stratified analysis showed a lower incidence of the MACE among men in the intervention group compared to control group (IRR 0.09 (95% CI [0.01 – 0.66]), and a tendency towards better effect of the intervention in patients aged 65 years or older, see Fig. 4.

### Prevention of chest pain (secondary endpoint)

Chest pain was reported in 19 patients of whom six patients (6/95, 6.3%) were in the intervention group and 13 (13/97, 13.4%) were in the control group, Fig. 2. Of these, 10 events were classified as angina with nine occurring in the control group. The incidence rate in the intervention group was 135 patients per 1,000 person-years and 308 patients per 1,000 person-years in the control group yielding an IRR of 0.44 (95% CI [0.14 – 1.23]).

### Association between CAC score and chest pain (tertiary endpoint)

**Fig. 5:**
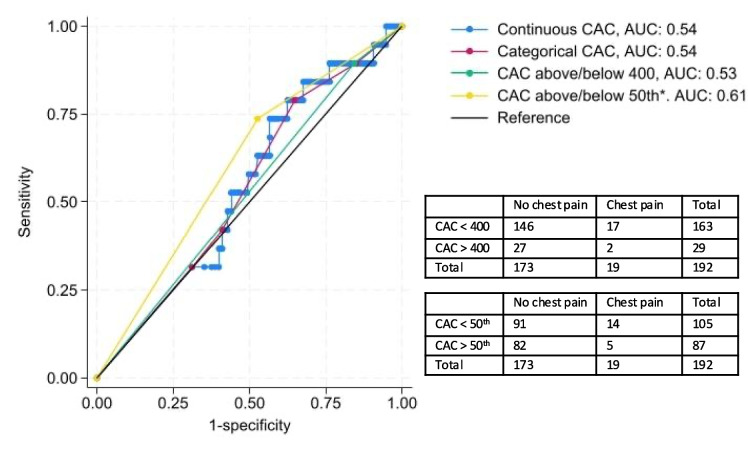
Discriminatory abilities of CAC score. Caption: Receiver-Operating Characteristic analyses of the discriminatory properties of coronary artery calcium score to identify fluoropyrimidines-induced chest pain in cancer patients and exact prevalence of events. Continuous CAC: Coronary artery calcium score used as a continuous scale. Categorical CAC: Coronary artery calcium score in clinical categories (0, 10, 100, 400). CAC above/below 400: Coronary artery calcium score dichotomized as above or below 400. CAC above/below 50th: Coronary artery calcium score dichotomized as above or below the age- and sex matched 50th percentile. Abbreviations: CAC: Coronary artery calcium

The median age- and sex adjusted CAC score was 42 (IQR [0–78]) for the total population. The median adjusted CAC score among patients with chest pain was 35 (IQR [0–54]) versus 47 (IQR [0–78]) among patients without chest pain. The discriminatory ability of the CAC score to predict the development of chest pain ranged from AUC 0.53 (95% CI [0.45 – 0.60]) to 0.61 (95% CI [0.50 – 0.71]) for the entire cohort, depending on whether the CAC score was dichotomized (at 400 or the 50th percentile) or used as a categorical (0, 10, 100, 400) or continuous variable, see Fig. 5. Sensitivity analyses excluding patients who received one or more cardiological interventions did not improve the discrimination ability.

## Discussion

This randomized controlled trial aimed to evaluate the potential benefits of systematic assessment and management of cardiovascular risk factors in cardiac asymptomatic patients prior to FP treatment. The follow-up period of six months was intentionally chosen to capture acute cardiotoxic events occurring during or shortly after chemotherapy administration. These early events are of particular clinical relevance, as they may lead to premature discontinuation of an effective oncologic treatment for which no equivalent alternatives are available. Consequently, our study did not aim to assess long-term cardiovascular outcomes, but rather to evaluate the feasibility of preventing treatment-related cardiotoxicity within the critical window of chemotherapy exposure. Furthermore, we aimed to investigate the predictive value of CAC score for the development of FP-induced chest pain in these patients. We found an IRR for MACE of 0.57, a point estimate indicating a potential reduction in events in the intervention group, although this did not reach statistical significance, and the confidence interval was wide. However, despite the small sample size, this study highlights the potential for prevention of FP-induced cardiotoxicity and provides valuable insights for designing and powering future studies.

### Prevention of FP-induced cardiotoxicity

Strategies to prevent cardiotoxicity have been explored in non-FP oncological regimens, primarily targeting reductions in left ventricular ejection fraction (LVEF) associated with anthracycline-based therapies through beta-blockers or ACE-inhibitors/angiotensin II receptor antagonists [19]. While attenuated LVEF declines have been observed, no differences were reported at extended follow-up overall [19]. Similarly, in the PREVENT-study, daily atorvastatin during anthracycline-based treatment did not affect declines in LVEF [26]. These preventive strategies mainly relied on single-agent pharmacological interventions rather than the comprehensive cardiovascular management recommended by current guidelines.

FP have received less attention despite their well-described cardiotoxic effects. Retrospective data from Zafar et al. demonstrated that secondary prophylaxis with nitrates or calcium channel blockers in patient with 5-FU-induced coronary vasospasm enabled the continuation of 5-FU therapy. However, 30% of patients with vasospasms discontinued 5-FU treatment [27]. Whether discontinuation could have been avoided through primary prevention remains unknown. The optimal choice of drug has not been established; beta-blockers are used for angina but not vasospasm, and nitrates or calcium channel blockers are also antianginal drugs. However, our study was not designed to investigate the mechanisms behind FP-induced chest pain or optimal treatment regimen, but therapeutic decisions were at the treating cardiologist’s discretion based on an individual patient assessment. Our study does propose a feasible and safe approach to primary prevention integrating guideline-informed cardiac care into the clinical pathway for cancer patients scheduled for FP-treatment. Studies with similar multidisciplinary management approach in breast cancer patients (NCT02571894) and elderly patients (NCT03711110) are ongoing.

Primary prevention of cardiotoxicity due to antineoplastic treatment has been considered preferable [28, 29], and it is recommended, that all patients undergo cardiovascular risk assessment and optimization before initiating cardiotoxic cancer therapy [16, 30]. However, cardiovascular risk factors have not been consistently identified as predictors of cardiotoxicity in FP-regimens, complicating the ability to predict which patient will develop cardiotoxic effects. Zafar et al. reported that patients who developed 5-FU-induced vasospasms were less likely to have any cardiovascular risk factors [12], and Lombardi et al. found no association between FP-induced cardiotoxicity and pre-existing cardiological comorbidities [14]. Our study highlights the potential for effective preventive strategies in patients without prior cardiac disease or symptoms. The intervention was feasible, with only eight patients not completing the cardiological assessment and CT scan before starting chemotherapy. In addition, explorative analyses suggest that elderly patients and men may benefit more from these interventions. These stratified analyses were exploratory in nature and should be interpreted with caution given the limited number of events and the absence of statistical power to detect subgroup effects.

Most cardiological interventions in our study were based on LDL measurements and a thorough assessment of lifestyle risk factors, with fewer interventions initiated based on TTE or ECG findings. These observations indicate that blood tests and anamnestic evaluations should be prioritized in preventive strategies for FP-induced cardiotoxicity.

### Predictive value of CAC score

Although the CAC score is a validated marker of coronary atherosclerosis which has been associated with an increased risk of coronary vasospasm [31], its role in predicting FP-induced cardiotoxicity has not been fully investigated. The relatively poor performance observed (AUCs 0.53–0.61) may therefore reflect both pathophysiological differences and the limited sample size.

Gal et al. demonstrated that a CAC score above 400 was associated with a higher cumulative incidence of acute coronary events among breast cancer survivors, particularly in those treated with anthracyclines and radiation therapy, after a median follow-up of 51 months [32]. In contrast, we did not find the CAC score to predict the risk of cardiotoxicity in cancer patients receiving FP treatment, likely due to the substantially shorter follow-up period. The average CAC score was below the expected age- and sex-adjusted median, and CAC score did not differ between patients reporting chest pain versus no chest pain. The difference between the studies may be attributed to distinct pathophysiological mechanisms of different treatment modalities affecting the coronary arteries. Further studies evaluating the risk of coronary events in cancer treatment might benefit from larger sample sizes and the use of CT coronary angiography.

### Strengths and limitations

This clinically relevant, randomized controlled single-center trial demonstrates high internal validity for exploring feasibility and suggesting relevant subgroups. The primary limitation is the insufficient sample size, constrained by limited resources, which may have hindered the ability to achieve statistical significance for the study endpoints. This limitation increases the risk of type 2 errors, and findings and generalizability should be interpreted with this limitation in mind. Some patients had prior chest radiation and anthracycline exposure which are risk-factors for long-term cardiovascular disease. However, due well-balanced distribution between study arms and low number of events, subgroup analyses would have been underpowered and potentially misleading. Detailed data on specific cardiovascular medications were not systematically collected as the study did not aim to test the efficacy of specific pharmacological agents. The study was non-blinded, which may have introduced detection bias, particularly regarding the reporting and interpretation of subjective symptoms such as chest pain. Although randomization helps mitigate systematic confounding, increased clinical attention in the intervention group may have influenced symptom awareness or health-seeking behavior. Additionally, risk assessment for cardiotoxicity was conducted using non-contrast CT scans. While this approach provides a CAC score, it does not account for significant stenosis or non-calcified plaques, both of which are important substrates for coronary events. A CT coronary angiography, in conjunction with the CAC score, could have provided a more comprehensive evaluation of cardiac risk. The non-contrast CT scan offers several advantages being faster and less expensive and exposes the patient to less radiation and no contrast and might therefore be more implementable in future trials and clinical practices within cardiotoxicity.

## Conclusions

In this randomized, controlled trial, we demonstrated that cardiological management of cardiovascular risk factors prior to treatment with fluoropyrimidines resulted in half as many cardiotoxic events, but the study did not reach statistical significance. Coronary artery calcium score did not predict chest pain. Further studies are needed to investigate the optimal strategies to prevent fluoropyrimidine-induced cardiotoxicity in cancer patients.

## Supplementary Information

Supplementary Material 1.

## Data Availability

The datasets used and/or analysed during the current study are available from the corresponding author on reasonable request.

## Abbreviations

5-FU: 5-Fluorouracil
ACS: Acute coronary syndrome
AUC: Area under the curve
CAC: Coronary artery calcium
CAG: Coronary arteriography
CT: Computed tomography
ECG: Electrocardiogram
FP: Fluoropyrimidines
IRR: Incidence rate ratio
LDL: Low-density lipoprotein
LVEF: Left ventricular ejection fraction
MACE: Major adverse cardiovascular events
NSTEMI: Non-ST elevation myocardial infarction
ROC: Receiver-operating characteristic
STEMI: ST elevation myocardial infarction
TTE: Transthoracic echocardiography
UAP: Unstable angina pectoris
