# Supplementary material for: Primary prevention of cardiotoxicity in cancer patients treated with fluoropyrimidines: a randomized controlled trial
Source: Cardiooncology. 2025 May 17;11:48. doi: 10.1186/s40959-025-00344-3 (PMC12084922; doi:10.1186/s40959-025-00344-3)
Supplement: Supplementary file 1 — Supplementary Material 1. [file 40959_2025_344_MOESM1_ESM.docx]

Supplementary File

“Primary prevention of cardiotoxicity in cancer patients treated with fluoropyrimidines: a randomized controlled trial”

Table 1. Study schedule

|  | STUDY PERIOD | | | | |
| --- | --- | --- | --- | --- | --- |
|  | Enrolment | Allocation | Intervention | Events | Follow-up at 6 mo. |
| Eligibiligy screen  Informed consent  Non-contrast cardiac CT  Blood pressure measurement  Electrocardiogram  Routine laboratory tests  Comorbidity  Smoking status  Randomization | X  X  X  X  X X X X | X |  |  | X  X  X  X |
| Cardiological assessment  Transthoracic echocardiography  Interventions according to Table 1 |  |  | X  X  X |  |  |
| Cardiac symptoms  Electrocardiogram  Laboratory tests  Treatment according to guidelines  Determination of urgency |  |  |  | X  X  X X X |  |
| Physical examination  Questionnaire (unreported cardiac symptoms) |  |  |  |  | X  X |

| Table 2. Detailed overview of primary endpoint | | | |
| --- | --- | --- | --- |
|  | Number of events in the intervention group (n=95) | Number of events in the control group (n=97) | Total number of events |
| Total number of events | 9 | 15 | 24 |
| Overall mortality   Cardiac mortality   Cancer-related mortaliry | 0 4 | 2  8 | 14 |
| Chest pain  Hospital admission  ACS with KAG | 3 2 | 5 | 10 |
